# Plant Regeneration and Somatic Embryogenesis from Immature Embryos Derived through Interspecific Hybridization among Different *Carica* Species

**DOI:** 10.3390/ijms131217065

**Published:** 2012-12-12

**Authors:** Md. Abul Kalam Azad, Md. Golam Rabbani, Latifah Amin

**Affiliations:** 1 Centre for General Studies, Universiti Kebangsaan Malaysia, UKM Bangi 43600, Selangor, Malaysia; E-Mail: nilam@ukm.my; 2 Department of Agricultural Extension, Khamarbari, Farmgate, Dhaka 1215, Bangladesh; 3 Department of Horticulture, Bangladesh Agricultural University, Mymensingh 2202, Bangladesh; E-Mail: drmgrabbani@yahoo.com

**Keywords:** *Carica* species, hybridization, plant regeneration, somatic embryogenesis

## Abstract

Plant regeneration and somatic embryogenesis through interspecific hybridization among different *Carica* species were studied for the development of a papaya ringspot virus-resistant variety. The maximum fruit sets were recorded from the cross of the native variety *C. papaya* cv. Shahi with the wild species *C. cauliflora*. The highest hybrid embryos were recorded at 90 days after pollination and the embryos were aborted at 150 days after pollination. The immature hybrid embryos were used for plant regeneration and somatic embryogenesis. The 90-day-old hybrid embryos from the cross of *C. papaya* cv. Shahi × *C. cauliflora* showed the highest percentage of germination, as well as plant regeneration on growth regulators free culture medium after 7 days pre-incubation on half-strength MS medium supplemented with 0.2 mg/L BAP, 0.5 mg/L NAA and 60 g/L sucrose. The 90-day-old hybrid embryos from the cross of *C. papaya* cv. Shahi × *C. cauliflora* produced maximum callus, as well as somatic embryos when cultured on half-strength MS medium containing 5 mg/L 2,4-D, 100 mg/L glutamine, 100 mg/L casein hydrolysate and 60 g/L sucrose. The somatic embryos were transferred into half-strength MS medium containing 0.5 mg/L BAP and 0.2 mg/L NAA and 60 g/L sucrose for maturation. The highest number of regenerated plants per hybrid embryo (10.33) was recorded from the cross of *C. papaya* cv. Shahi × *C. cauliflora*. Isoenzyme and dendrogram cluster analysis using UPGMA of the regenerated F_1_ plantlets confirmed the presence of the hybrid plantlets.

## 1. Introduction

Papaya (*Carica papaya* L) is a tropical fruit having commercial importance because of its high nutritive and medicinal value. It is a quick-growing fruit and available year-round. Ripe fruits contain 10 percent sugar and are rich in different minerals and vitamins, particularly vitamins A and C. The latex of papaya is the source of two proteolytic enzymes such as papain and chymopapain [1]. The origin of the papaya is South Mexico and Costa Rica [2]. Most of the papaya-cultivating countries are India, Brazil, Mexico, Nigeria, Indonesia, China, Peru, Thailand and the Philippines [3]. Papaya cultivation is affected by a number of diseases caused by various pathogens and viruses. The most destructive disease of *C. papaya* worldwide is papaya ringspot, caused by papaya ringspot virus-type P (PRSV-P) [4,5]. Typical symptoms of papaya ringspot virus appear as the ringspot on the fruit, a water-soaked lesion on the petioles and stem, and chlorosis or mottle on the leaves. The seedlings infected by the papaya ringspot virus seldom reach maturity, and severely infected adult plants decline rapidly and fail to produce fruit [5]. It deteriorates the fruit quality and makes the fruits unfit for human consumption. Control measures used against PRSV-P include cultural practices, cross-protection, and planting of tolerant cultivars [6]. None of these has been very successful, and development of virus-resistant cultivar is the most reliable solution for long-term control. None of the *C. papaya* cultivars tested was naturally resistant to PRSV-P [7]. A monogenic dominant resistant gene to PRSV-P exists in some wild but related species of papaya such as *C. cauliflora*, *C. stipulata* and *C. pubescens*[8]. Conventional interspecific hybridization of *C. papaya* with these species has been difficult because of interspecific reproductive barriers [9]. Interspecific hybrid plants obtained from the cross between *C. papaya* and *C. cauliflora* did not survive after transfer to a sterile medium [8]. On the other hand, fertile interspecific hybrids and F_2_ population were obtained from the cross between *C. papaya* and *C. cauliflora* through embryo rescue technique [10]. Postzygotic disruption is the main problem in interspecific crosses of *C. papaya* and *C. cauliflora*, and embryos abort before differentiating into polyembryonic structures [9]. The success of interspecific hybridization between *C. papaya* and *C. cauliflora* depends upon the genotypes of both the species used in a hybridization program [9,11]. Interspecific hybrid embryos of *C. papaya* and *C. cauliflora* are known to abort during 90–120 days after pollination [9]. Consequently, rescued embryos are weak and difficult to establish *in vitro*[12]*. C. papaya* cv. Shahi and *C. papaya* cv. Ranchi are two popular local papaya varieties which are widely grown by farmers in Bangladesh. The first is a native variety, while *C. papaya* cv. Ranchi was first introduced in India. To date, there have been no published studies on interspecific hybridization between these two varieties with the wild species *C. cauliflora and C. goudotiana*, and there has been no attempt on interspecific hybridization for plant regeneration and somatic embryogenesis of their hybrids. Therefore, the present study has been undertaken to develop efficient and suitable techniques for plant regeneration and somatic embryogenesis with a view to develop a papaya ringspot virus-resistant variety through interspecific hybridization among *C. papaya* cv. Shahi, *C. papaya* cv. Ranchi, *C. cauliflora* and *C. goudotiana.*

## 2. Results and Discussion

### 2.1. Fruit Set and Embryo Rescue

The results of different crosses among *Carica* species and embryo formation are presented in Table 1. The cross of *C. papaya* cv. Shahi × *C. cauliflora* showed the highest percentage of fruit set (64.67%) followed by C*. papaya* cv. Shahi × *C. goudotiana* (59.73%) and *C. papaya* cv. Ranchi × *C. cauliflora* (58.10%). The lowest percentage of fruit set was recorded from the cross of C. *papaya* cv. Ranchi × *C. goudotiana* (54.36%). Embryo development was observed at 60, 90 and 120 days after pollination, and embryos were aborted at 150 days after pollination. The heart-shape stage of the embryo was noticed at 60 days after pollination, but was very weak. On the other hand, early cotyledonary and cotyledonary stage were noticed at 90 and 120 days, respectively, after pollination. The maximum number of embryos per fruit were from the cross of by *C. papaya* cv. Shahi × *C. goudotiana* (20.53) at 90 days after pollination which was statistically identical with *C. papaya* cv. Shahi × *C. cauliflora* (18.30) and *C. papaya* cv. Ranchi × *C. goudotiana* (17.07), and the lowest number of embryo per fruit was found in *C. papaya* cv. Ranchi × *C. cauliflora* (15.40). The number of embryos per fruit decreased at 120 days after pollination and these embryos were necrotic and deformed. No embryo was found at 150 days after pollination. These results indicate that *C. cauliflora* and *C. goudotiana* were sexually incompatible with *C. papaya* cv. Shahi and *C. papaya* cv. Ranchi due to embryo abortion before seed maturation. A similar result was reported by Manshardt and Wenslaff [9] where interspecific hybrid embryos of *C. papaya* × *C. cauliflora* have been found to be aborted 90–120 days after pollination

### 2.2. Plant Regeneration from 90-Day-Old Embryos

The fruits were harvested at 90 days after pollination. The hybrid embryos were rescued and pre-incubated for seven days on half-strength MS medium supplemented with 0.2 mg/L BAP and 0.5 mg/L NAA for germination. The embryos obtained from the crosses of *C. papaya* cv. Shahi × *C. cauliflora* performed at a good germination rate, with early shoot and root initiation, whereas the embryos obtained from the crosses of *C. papaya* cv. Ranchi × *C. goudotiana* showed poor percentage of germination and delayed shoot and root initiation (Table 2). The germinated embryos were transferred onto MS medium without growth regulators for growth and development. Significant variation with respect to plant height, number of leaves and roots per plant were observed (Table 2). The highest plant height was obtained from the immature embryos of *C. papaya* cv. Shahi × *C. cauliflora* (4.26 cm) and the lowest plant height was recorded from the cross of *C. papaya* cv. Ranchi × *C. goudotiana* (3.33 cm) which was statistically identical with the crosses between *C. papaya* cv. Ranchi × *C. cauliflora* (3.43 cm) and *C. papaya* cv. Shahi × *C. goudotiana* (3.50 cm). The embryo obtained from the crosses of *C. papaya* cv. Shahi × *C. cauliflora* produced the maximum number of leaves (5.60) which was statistically identical with *C. papaya* cv. Shahi × *C. goudotiana* and *C. papaya* cv. Ranchi × *C. cauliflora* while the lowest number of leaves (4.63) was regenerated from the embryo obtained from the crosses of *C. papaya* cv. Ranchi × *C. goudotiana.* On the other hand, the maximum number of roots (6.81) were recorded from embryos obtained from crosses of *C. papaya* cv. Shahi × *C. cauliflora* which was statistically identical with *C. papaya* cv. Ranchi × *C. cauliflora* and *C. papaya* cv. Shahi × *C. goudotiana* while the plantlets derived from crosses of *C. papaya* cv. Ranchi × *C. goudotiana* produced the lowest number of roots (5.78). These results revealed that immature hybrid zygotic embryo were able to produce plantlets and the hybrid embryos derived from *C. papaya* cv. Shahi × *C. cauliflora* was found to be the best among all the crosses. This is the first study on plantlet regeneration of immature embryo from these hybrids. These results are in agreement with Magdilita *et al.*[12] who reported that the immature embryo of another variety of *C. papaya* which has been crossed with *C. cauliflora* showed good plantlet regeneration.

### 2.3. Somatic Embryogenesis from 90-Day-Old Embryos

**Figure 1:**
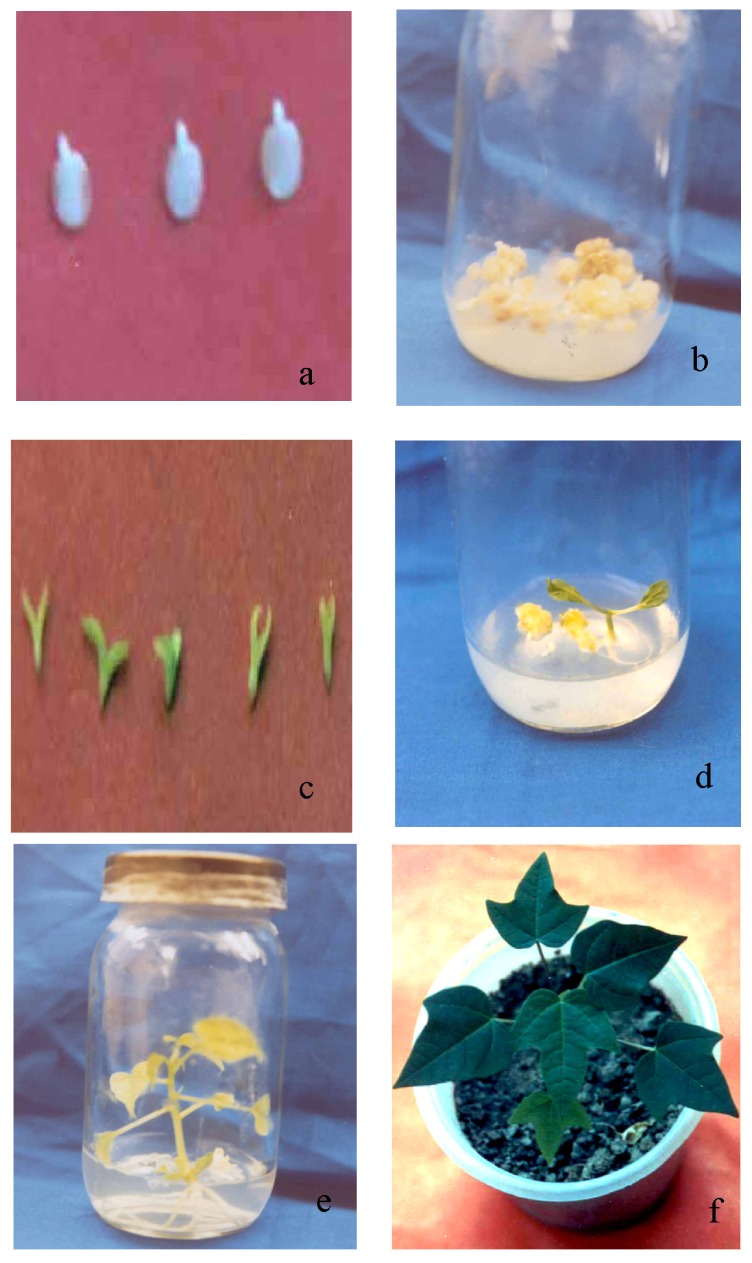
The somatic embryogenesis of immature zygotic hybrid embryos derived from the crosses of *C. papaya* cv. Shahi × *C. cauliflora* (**a**) Immature zygotic hybrid embryos from obtained at 90 days after pollination; (**b**) Profuse callus of immature hybrid embryos after culturing; (**c**) Mature somatic embryos; (**d**) Germinated somatic embryos; (**e**) Hybrid plant in the culture tube and (**f**) Acclimatized to *ex vitro* condition hybrid plant.

Rescued embryos were weak and very difficult to establish for plant regeneration. Immature hybrid embryos were cultured for somatic embryogenesis. The 90-day-old embryos obtained from interspecific hybridization of different *Carica* species were cultured onto half-strength MS medium supplemented with 5.0 mg/L 2,4-D, 100 mg/L glutamine, 100 mg/L casein hydrolysate and 60 g/L sucrose for callus induction. The highest percentage of callus (68.03%) were recorded from the cross of *C. papaya* cv. Shahi × *C. goudotiana* followed by *C. papaya* cv. Ranchi × *C. goudotiana* (64.40%) and lowest percentage of callus (54.80%) was recorded from *C. papaya* cv. Shahi × *C. cauliflora* (Table 3). This might be due to fact that immature embryos of *C. papaya* cv. Shahi × *C. cauliflora* have more callusing in nature, thus leading more response to somatic embryos. This result indicates that 2,4-D was suitable for somatic embryogenesis of *C. papaya* as recommended by earlier researchers [13,14]. The callus was transferred into culture medium without growth regulator for embryogenic callus and somatic embryos induction. Early callus and embryogenic callus formation was recorded from the embryos of *C. papaya* cv. Ranchi × *C. cauliflora* whereas delayed callusing and embryogenic callus was observed in case of *C. papaya* cv. Ranchi × *C. goudotiana*. The maximum number of somatic embryos (14.13) was recorded from the cross of *C. papaya* cv. Shahi × *C. cauliflora* and the lowest number of somatic embryos (9.80) was recorded from the cross of *C. papaya* cv. Shahi × *C. goudotiana.* The somatic embryos were transferred onto half-strength MS medium containing 0.5 mg/L BAP and 0.2 mg/L NAA and 60 g/L sucrose for maturation for four weeks. After maturation, the somatic embryos were transferred onto a hormone-free medium for plant regeneration. Somatic embryos of *C. papaya* cv. Shahi × *C. cauliflora* produced the highest number of plantlets (10.33) while the lowest number of plantlets (6.60) was found from somatic embryos of *C. papaya* cv. Shahi × *C. gouditiana.* The hybrid plantlets obtained from somatic embryogenesis are shown systematically in Figure 1. This might be due to differences among the genotypes. These results are in agreement with Moore and Litz [15], who reported the induction embryogenic zygotes in interspecific crosses between *C. papaya* and *C. cauliflora*. Malabadi *et al.*[16] reported that the ability to induce embryogenic tissue varied from different papaya varieties and the highest percentage of somatic embryogenesis (87.0%) was noticed in a papaya variety Taiwan-786. Ananda *et al.*[17] reported that zygotic embryos of *C. papaya* cv. Co7 derived from the white seed of 110–120-day-old fruits resulted in better embryogenic callus induction. Fitch and Manshardt [13] reported that genotype played a significant role in success of somatic embryogenesis and *C. papaya* cv. Sunset showed 93% embryonic yield on medium containing 5 mg/L 2,4-D. Our results of callus induction were slightly lower compared to Fitch and Manshardt [13] due to a different variety used in this study. Farzana *et al.*[18] reported that mature zygotic embryos of *C. papaya* cv. Rathna produced somatic embryos and, subsequently, plantlets. Somatic embryos of papaya produced higher cell mass after culturing on medium containing 4.97 mg/L 2,4-D and 0.66 mg/L ABA [19]. Litz and Conover [20] found the induction of somatic embryos of *C. papaya* on MS Medium containing 20% coconut water. Interspecific hybridization between *C. cauliflora* and *C. papaya* followed by immature embryo rescue for somatic embryogenesis has been cited as an effective method for PRSV-P gene transfer in papaya [21].

### 2.4. Isoenzyme Analysis

**Figure 2:**
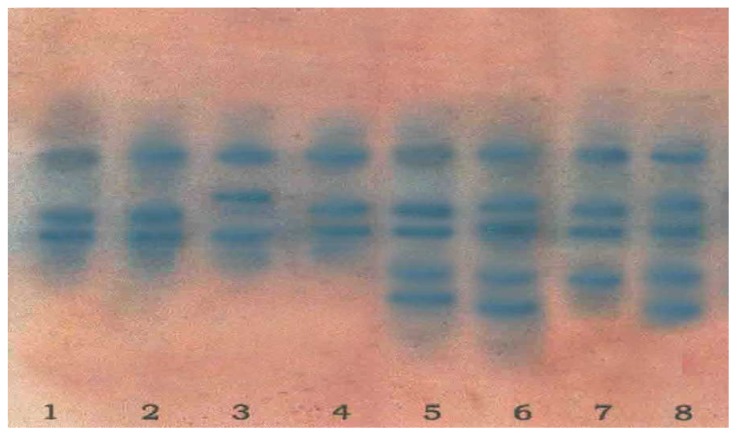
Isoenzyme analysis of Glutamate Oxaloacetate Transminase (GOT) of different *Carica* species and their hybrids. (Lane 1 = *C. papaya* cv. Shahi, lane 2 = *C. papaya* cv. Ranchi, lane 3 = *C. cauliflora*, lane 4 = *C. goudotiana*, lane 5 = hybrid of *C. papaya* cv. Shahi × *C. cauliflora*, lane 6 = hybrid of *C. papaya* cv. Ranchi × *C. cauliflora*, lane 7 = hybrid of *C. papaya* cv. Shahi × *C. goudotiana*, lane 8 = hybrid of *C. papaya* cv. Ranchi × *C. goudotiana.*

**Figure 3:**
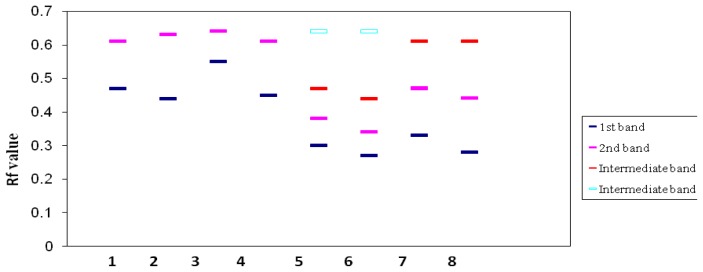
Zymogram of (GOT) isoenzyme of different *Carica* species and their hybrids. (Lane 1 = *C. papaya* cv. Shahi, lane 2 = *C. papaya* cv. Ranchi, lane 3 = *C. cauliflora*, lane 4 = *C. goudotiana*, lane 5 = hybrid of *C. papaya* cv. Shahi × *C. cauliflora*, lane 6 = hybrid of *C. papaya* cv. Ranchi × *C. cauliflora*, lane 7 = hybrid of *C. papaya* cv. Shahi × *C. goudotiana*, lane 8 = hybrid of *C. papaya* cv. Ranchi × *C. goudotiana*.

**Figure 4:**
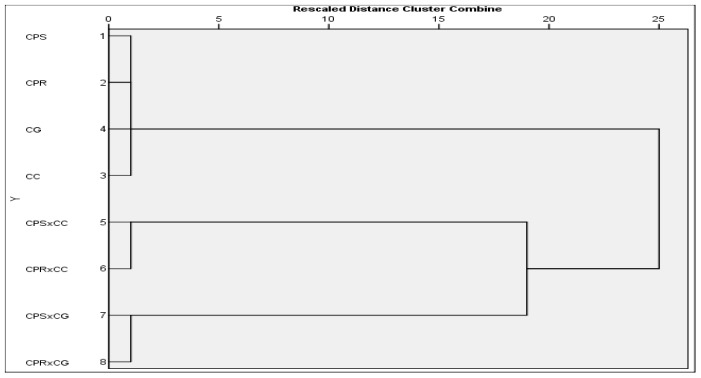
Dendrogram cluster analysis using UPGMA showing the relationship among the parents and their hybrids of different *Carica* species from GOT isoenzyme data (CPS = *C. papaya* cv. Shahi, CPR = *C. papaya* cv. Ranchi, CC = *C. cauliflora*, CG = *C. goudotiana*).

The Glutamate Oxaloacetate Transminase (GOT) isoenzyme and zymogram banding patterns of four parents and four interspecific hybrids are shown in Figures 2 and 3. The four parents *viz. C. papaya* cv. Shahi, *C. papaya* cv. Ranchi, *C. cauliflora* and *C. goudotiana* had two prominent monomeric bands. The hybrid plants obtained from the crosses of *C. papaya* cv. Shahi × *C. cauliflora* and *C. papaya* cv. Ranchi × *C. cauliflora* showed four bands which were two monomeric and two polymeric. The two bands corresponded to both parents and another two bands were intermediate and not found in either of the parents. The hybrid plants obtained from the crosses of *C. papaya* cv. Shahi × *C. goudotiana*, *C. papaya* cv. Ranchi × *C. goudotiana* showed three bands. Among the three bands, two bands corresponded to both the parents and one band was intermediate which was not found in either parents. These differences could be due to the differences in total protein concentration in the hybrid samples. Isoenzyme patterns of regenerated plantlets between *C. papaya* and *C. cauliflora* showed new bands which indicated hybrid plants [17]. The diversity of the banding pattern was seen from the Rf value. Zymogram protein-banding patterns can indicate differences between parents and hybrid plants in *Carica* species. Molecular characterization of genetic variations should be inferred from theprotein-banding pattern because it produces more accurate data since protein is the product of late gene expression and cannot be easily changed. The results in Figures 2 and 3 showed that there were striking differences between the parents of *C. papaya* cv. Shahi, *C. papaya* cv. Ranchi. *C. cauliflora*, *C. goudotiana* and their hybrids on the level of molecular characteristics. Dendrogram clustering analysis using UPGMA is shown in Figure 4. From the cluster analysis, *C. papaya* cv. Shahi and *C. papaya* cv. Ranchi were clearly separated from *C. cauliflora* and *C. goudotiana*. The dendrogram using UPGMA demonstrated that all hybrids are distinct differences from their parents.

## 3. Experimental Section

### 3.1. Plant Materials and Hybridization Procedure

*Carica papaya* cv. Shahi and *Carica papaya* cv. Ranchi are dioecious local varieties. The first is a native species which was released in Bangladesh in the year 1992 by Bangladesh Agricultural Research Institute, Joydebpur Gazipur, Bangladesh, while *C. papaya* cv. Ranchi was first introduced in India. *Carica cauliflora* and *Carica goudotiana* are the wild species of *Carica*. A monogenic dominant resistant gene to PRSV-P exists in *C. cauliflora*[8] and a phytophthora resistance gene exists in *Carica goudotiana*[22]. Crosses were made among the different *Carica* species. *C. papaya* cv. Shahi and *C. papaya* cv. Ranchi were selected as female plants while *C. caulifora* and *C. goudotiana* were selected as male plants during interspecific hybridization. Initially, the flowers which were expected to open in the next day were selected for pollination. The female flowers were bagged with perforated waxy paper in the afternoon. At the time of bagging, the open female flowers were removed to avoid any pollen contamination. The profusely dehiscent male flowers were picked during pollination. The petals of male flowers were removed and the pollens were dusted onto the wet stigmas during the day of anthesis of female flowers. The crossing was done in between 8 a.m. and 10 a.m. The waxy paper bag was replaced after hand pollination. The bag was removed 5–7 days after pollination when the fruits were set and the stigma dried up. The fruits were harvested at 60, 90, 120 and 150 days after pollination and were cut with the help of knife to examine for the presence of embryos in the ovules.

### 3.2. Plant Regeneration and Somatic Embryogenesis

The fruits were harvested at 90 days after pollination. The fruits were washed thoroughly under running tap water with a few drops of liquid soap. The fruits were then surface sterilized with 70% ethanol for 1 min and rinsed three times with sterilized distilled water and subsequently sterilized with 0.1% HgCl_2_ solution containing few drops of “Tween 80” for 15 min. Immature hybrid embryos at the cotyledonary stage were used for plant regeneration and somatic embryogenesis. The embryos were separated from the ovules by cutting with the help of pair of forceps and were ready for inoculation. The culture medium is one of the most important factors for plant regeneration and somatic embryogenesis. The Murashige and Skoog [23] medium was used through out the study. Half-strength MS medium supplemented with 0.2 mg/L BAP and 0.5 mg/L NAA were used for germination as recommended by Magdilita *et al.*[12]. After germination, growth regulator-free half-strength MS medium was used for plant growth and development. 2,4-D growth regulator has been commonly and successfully used for somatic embryogenesis of mature zygotic embryo [13,14]. In this study, 5 mg/L 2,4-D as well as 100 mg/L glutamine, 100 mg/L casein hydrolysate and 60 g/L sucrose was added to half-strength MS for callus induction as recommended by Fitch and Manshardt [13] and Teixeira da Silva *et al.*[14]. The callus was transferred onto growth regulator-free MS medium for embryogenic callus and somatic embryos development. The somatic embryos were transferred onto half strength MS medium containing 0.5 mg/L BAP and 0.2 mg/L NAA and 60 g/L sucrose for maturation as recommended by Ananda *et al.*[17] with slight modification. The cultures were kept in 16 h photoperiod at 24 °C ± 2 °C in a growth room. After callusing and germination, all cultures were kept at 25 °C illuminated with cool light from 1.83 m fluorescent tubes (4.83 ft. C 84 TDL/Philips). These tubes gave a broad spectrum of light, especially in red wavelength, which promoted growth and development.

### 3.3. Acclimatization of Plantlets

Well-developed plantlets were removed from culture and washed in sterilized water to remove traces of the medium. These plantlets were transplanted to plastic pot containing a mixture of autoclaved cocopeat, sand and garden soil (1:1:1). The plants were grown in growth chambers (24 °C ± 2 °C and 16 h photoperiod with 80% relative humidity). The plants were sprayed with Hoagland solution once a week. After two weeks, the plants were transferred to the greenhouse. Seventy-five percent od rhw plantlets were successfully transferred into the pot.

### 3.4. Isoenzyme Analysis

Vertical polyacrylamide gel electrophoresis was used for isoenzyme analysis. The uniform seedlings of hybrid and parent plants were selected for isoenzyme study. The leaves of the seedlings were weighed immediately after harvest and frozen in pre-cooled mortar (4 °C) kept on ice. 100 mg of fresh tissue of each sample was ground and homogenized in a chilled mortar by adding 50 mg of polyclar AT and 1.0 mL of extraction buffer in the presence of acid washed sand (pH 5.4). Extraction buffer consisted of 0.24% Tris and 5% sucrose. Then the sample was transferred to microtubes and centrifuged at 14,000 × *g* for 20 min at 1 °C. The gels were prepared from the stock solution of Acrylamide (29.2 g), Bis (0.8 g), Tris (18.17 g) and ammonium per sulfate (0.1 g) by weight and were dissolved in water and the pH was adjusted to 8.8 and finally the volume was made up to 100 mL by adding distilled water. The electrode buffer was prepared by dissolving Tris (1.2 g) and glycine (5.8 g) in about 150 mL distilled water and the volume was made up to 200 mL and diluted 10 times for use

The samples for electrophoresis were thoroughly mixed with 5 μL bromophenol blue (BPB) in sucrose (1% BPB in 25% sucrose solution) before being loaded into the gel. The vertical polyacrylamide gel electrophoresis (PAGE) was carried out at a constant current of 220 volt and at 4 °C for 4.5 h. For the staining, GOT was used, as recommended by Jobin-Decor *et al.*[24]. First AAT (pH 7.4) solution was prepared by mixing 800 mL water, α-Ketoglutaric acid (292 mg), L-Aspertic acid (1.07 g), PVP-40 (polyvinyl pyrrolidonel) (4.0 g), EDTA Na_2_ salt (400 mg) and Sodium phosphate dibasic (11.36 gm). Then 50 mg fast blue BB salt was added to the substrate solution. The gels were then incubated in the mixed solution at room temperature in the dark until the blue band appeared in the gels. The gels were washed thoroughly and fixed with 50% glycol, sealed with polythene and stored in the refrigerator. Isoenzyme-banding patterns were recorded on the basis of number and relative front (Rf) values of the bands. The Rf value is the mobility of each isoenzyme band that traveled from the origin divided by the distance traveled by the front tracking dye.

### 3.5. Data Collection and Analysis

The complete randomized design was used for all experiments. For each treatment, 60 embryos were cultured (12 embryos per culture tube and five replicates per treatment) and the experiment was repeated three times. The cultures were observed periodically and morphological changes were recorded at regular intervals. Cluster analysis was performed using unweighted pair-group method arithmetic average (UPGMA) and dendrogram indicating genetic similarities was constructed. Data were recorded and analyzed using SAS statistical package and comparison of the mean values across hybrids were analyzed using Duncan’s multiple range test.

## 4. Conclusions

Plant regeneration and somatic embryogenesis through interspecific hybridization between the local papaya varieties and the wild papaya species have been successfully carried out. The cross between the native variety *C. papaya* cv. Shahi × *C. cauliflora* yielded the maximum number of fruits. The immature 90-day-old hybrid embryo of *C. papaya* cv. Shahi × *C. cauliflora* showed the highest percentage of germination, as well as plant regeneration. The embryos at 90 days after pollination were successfully cultured on half-strength MS medium containing 5.0 mg L^−1^ 2,4-D, 100 mg L^−1^ glutamine, 100 mg L^−1^ casein hydrolysate and 60 gm L^−1^ sucrose for somatic embryogenesis. The embryos from the cross of *C. papaya* cv. Shahi × *C. cauliflora* also produced the highest number of plantlets. Isoenzyme and dendrogram using UPGMA clustering analysis confirmed that all F_1_ plantlets obtained through the interspecific hybridization were hybrids. These hybrids can be used by researchers, academicians and plant breeders to transfer the PRSV-P gene from the wild papaya species to native cultivars of papaya to develop a papaya ringspot virus-resistant variety.

## Figures and Tables

**Table 1:** Percentage of fruit set and embryo formation after interspecific hybridization of *C. papaya* cv. Shahi, *C. papaya* cv. Ranchi, *C. cauliflora* and *C. goudotiana.*

Cross	Fruit set (%) (Mean ± SE)	Number of embryos per fruit			

		60 days after pollination (Mean ± SE)	90 days after pollination (Mean ± SE)	120 days after pollination (Mean ± SE)	150 days after pollination (Mean ± SE)
CPS × CC	64.67 ± 2.27 a	13.33 ± 0.63 bc	18.30 ± 0.55ab	3.30 ± 0.40 a	0.00 ± 0.00 a
CPS × CG	59.73 ± 2.17 b	18.36 ± 1.03 a	20.53 ± 0.84 a	3.76 ± 0.21 a	0.00 ± 0.00 a
CPR × CC	58.10 ± 1.36 b	11.70 ± 0.94 c	15.40 ± 1.07 b	1.93 ± 0.37 b	0.00 ± 0.00 a
CPR × CG	54.36 ± 2.04 c	15.43 ± 0.41 b	17.07 ± 1.04 ab	2.37 ± 0.17 b	0.00 ± 0.00 a

CPS = *C. papaya* cv. Shahi, CPR = *C. papaya* cv. Ranchi, CC = *C. cauliflora* and CG = *C. goudotiana*, Means with different letters denote a significantly difference at *p* < 0.01 as determined by Duncan’s multiple range test.

**Table 2:** Plant regeneration from 90-day-old hybrid embryos obtained from interspecific hybridization of *C. papaya* cv. Shahi, *C. papaya* cv. Ranchi, *C. cauliflora* and *C. goudotiana*.

Cross	Germination (%) (Mean ± SE)	Days to shooting (Mean ± SE)	Days to rooting (Mean ± SE)	Plant height (cm) (Mean ± SE)	No. of leaves per plant (Mean ± SE)	No. of roots per plant (Mean ± SE)
CPS × CC	74.13 ± 2.25 a	13.47 ± 0.61 c	17.23 ± 0.83 b	4.26 ± 0.17 a	5.60 ± 0.17 a	6.81 ± 0.24 a
CPS × CG	5.80 ± 1.99 b	16.57 ± 0.57 ab	19.50 ± 0.63 ab	3.50 ± 0.17 b	5.50 ± 0.17 a	6.13 ± 0.20 a
CPR × CC	62.43 ± 1.64 b	14.87 ± 0.82 bc	17.47 ± 0.72 b	3.43 ± 0.18 b	5.43 ± 0.23 a	6.48 ± 0.21 a
CPR × CG	60.67 ± 1.04 b	18.27 ± 0.54 a	20.70 ± 0.60 a	3.33 ± 0.08 b	4.63 ± 0.08 b	5.78 ± 0.09 bc

CPS = *C. papaya* cv. Shahi, CPR = *C. papaya* cv. Ranchi, CC = *C. cauliflora* and CG = *C. goudotiana*, Means with different letters denote a significantly difference at *p* < 0.01 as determined by Duncan’s multiple range test.

**Table 3:** Somatic embryogenesis of 90-day-old hybrid embryos obtained from interspecific hybridization of *C. papaya* cv. Shahi, *C. papaya* cv. Ranchi, *C. cauliflora* and *C. goudotiana* cultured on half-strength MS medium containing 5 mg/L 2,4-D, 100 mg/L glutamine, 100 mg/L casein hydrolysate and 60 g/L sucrose.

Cross	Hybrid embryo producing callus (%) (Mean ± SE)	Days required for callusing (Mean ± SE)	Hybrid embryo producing embryogenic callus (%) (Mean ± SE)	Days required for embryogenic callus (Mean ± SE)	Number of somatic embryo per hybrid embryo (Mean ± SE)	Number of plantlets per hybrid embryo (Mean ± SE)
CPS × CC	68.03 ± 1.46 a	18.30 ± 0.60 a	48.37 ± 2.22 a	38.43 ± 1.01 a	14.13 ± 0.40 a	10.33 ± 0.52 a
CPS × CG	54.80 ± 2.23 c	15.47 ± 0.49 b	38.40 ± 1.21 c	29.47 ± 0.63 c	9.80 ± 0.23 c	6.60 ± 0.51 bc
CPR × CC	64.40 ± 1.15 ab	17.10 ± 0.62 ab	47.20 ± 1.80 ab	33.63 ± 178 b	12.57 ± 1.21 ab	9.41 ± 0.61 a
CPR × CG	60.80 ± 1.43 b	16.56 ± 0.52 ab	41.47 ± 1.92 bc	31.10 ± 0.81 bc	11.40 ± 0.69 bc	8.37 ± 0.52 ab

CPS = *C. papaya* cv. Shahi, CPR = *C. papaya* cv. Ranchi, CC = *C. cauliflora* and CG = *C. goudotiana*, Means with different letters denote a significantly difference at *p* < 0.01 as determined by Duncan’s multiple range test.
